# Attention to COVID 19 pandemic resulted in increased measles cases and deaths in Zambia

**DOI:** 10.1186/s41182-025-00736-2

**Published:** 2025-04-25

**Authors:** Kelvin Mwangilwa, Cephas Sialubanje, Musole Chipoya, Chilufya Mulenga, Moses Mwale, Charles Chileshe, Danny Sinyange, Moses Banda, Priscilla Nkonde Gardner, Lilian Lamba, Precious Kalubula, John Simwanza, Davie Simwaba, Nathan Kapata, Jonathan Mwanza, Peter J. Chipimo, Nyuma Mbewe, Nyambe Sinyange, Isaac Fwemba, Muzala Kapin’a, Roma Chilengi

**Affiliations:** 1 Zambia National Public Health Institute, Stand Number 1186 Corner of Chaholi Road and Addis Ababa, Rhodes Park, Lusaka, Zambia; 2 WHO Country Office, PO Box 32346, 10101 Lusaka, Zambia; 3 Department of Health Policy, Systems and Management, School of Public Health, University of Zambia, Ridgeway Campus, P.O. Box 50110, Lusaka, Zambia

**Keywords:** COVID-19 pandemic, Childhood, Routine immunization, Measles mortality, Measles vaccination

## Abstract

**Background:**

The COVID-19 pandemic had a devastating impact on childhood routine immunization programs, resulting in increased measles mortalities and complications. In Zambia, the likelihood of measles-related deaths and complications in children was possibly increased because of high rates of unvaccinated children, late diagnosis, and poor case management, which could have been a consequence of exclusive focus on COVID-19 interventions. This study aimed at examining the effect of the COVID-19 pandemic on measles mortality and its predictors among patients seen at health facilities in Zambia.

**Methods:**

We used longitudinal data (January 2020 to August 2023) from outbreak investigations and time series data from 2017 to 2023 to understand the impact of COVID-19 on measles immunization and know the predictors of measles mortalities. The period running from January 2017 to February 2020, just before the first reported COVID-19 case, was defined as pre-COVID-19, and March 2020 to December 2023 as post-COVID-19. Multivariable logistic regression analysis was used to determine predictors of mortality. A segmented Poisson regression model was used to determine the correlation between the underlying patterns of measles mortality and the commencement of the COVID-19 pandemic.

**Results:**

A total of 3429 measles cases were reported during the study period. Of these, 1261 had complete metadata and were included in the analysis. The median age was 3 years (IQR, 1–7). Out of the 1261 enrolled, 54 (4.3%) were reported died. A total of 205 (21.0%) were IgM positive, and 207 (16.9%) were vaccinated. Monthly measles mortality increased by 220%, from 0.06 per 100,000 before COVID-19 to 0.23 during the pandemic. Predictors of mortality were younger age category (0–4) (AOR = 2.78; 95% CI 1.16–7.14), testing positive for measles IgM (AOR = 2.17; 95% CI 1.07–4.39), rush (AOR = 3.66; 95% CI 1.31, 6.21), and female sex (AOR = 1.90; 95% CI 1.04–3.50), which increased the odds of dying. However, being vaccinated (AOR = 0.06; 95% CI 0.01–0.42) reduced the odds of dying. Evidence for the COVID-19 effect was strongly associated with increased measles mortality (RR, 1.02; 95% CI 1.00, 1.04; 0.017) with a trend step change of 81% (RR, 1.81; 95% CI 1.14–2.87). There was also an increased trend of measles cases (RR, 1.04; 95% CI 1.01–1.06) during the pandemic. Measles dose 2 vaccination trends increased by about 0.3% during the COVID-19 pandemic due to the Supplementary Immunization Activity (SIA) (RR, 1.003; 95% CI 1.000–1.010). However, there was a dramatic drop of about 42% (RR = 0.58, 95% CI 0.46–0.72).

**Conclusions:**

Measles caused a significant increase in child mortality during the pandemic period. A mix of systemic, clinical, and individual factors affected measles mortality. Prioritizing vaccine coverage, especially for younger children and marginalized populations; enhancing diagnostic and treatment capacities; and addressing gender and healthcare access disparities are all essential components of interventions aimed at lowering mortality. The findings suggest that public health interventions focusing on measles vaccination, rapid detection, and appropriate case management are crucial to reducing mortality and preventing further transmission. To achieve population immunity, sustained efforts are required to maintain high coverage rates.

## Background

Measles is one of the most contagious illnesses in the world, and herd immunity requires high vaccination rates (around 95%) [1]. However, vaccination services were hampered globally by the COVID-19 epidemic. The World Health Organization (WHO) reports that more than 23 million children did not receive their recommended immunizations in 2020, which is the most since 2009 [2]. It has been suggested that a number of circumstances contributed to this gap, such as limited access to medical facilities, movement restrictions, and the redirection of healthcare resources to address COVID-19 [2]. The drop in vaccination rates was further exacerbated by communities'reluctance to visit healthcare facilities, because they were afraid of catching COVID-19 [3]. Global health services were significantly affected by the COVID-19 pandemic, which forced resources to be diverted to manage the outbreak, while routine immunization efforts experienced delays [4]. According to a WHO pulse survey conducted in May and June 2020 that was focused on maintaining the continuity of essential health services during the COVID-19 pandemic, routine immunization was one of the most disrupted services in comparison with other critical health services [5]. Guidelines for immunization efforts during the COVID-19 pandemic were released by the World Health Organization (WHO) in March 2020 [6]. According to a WHO pulse survey conducted in May and June 2020 that was focused on maintaining the continuity of essential health services during the COVID-19 pandemic, routine immunization was one of the most disrupted services in comparison with other critical health services [5]. In 2020, there were 26 countries with significant measles outbreaks, which accounted for 84% of all cases reported. It has been reported that the likelihood of measles-related deaths and significant complications in children is increased by high rates of unvaccinated children, measles outbreaks, and disease detection and diagnostics redirected to support COVID-19 interventions [7, 8]. Similarly, historical data from past infectious threats, wars, and global disease outbreaks has demonstrated that gaps in primary care access and a focus away from routine medical care increase morbidity and mortality [9]. The future of a long-fought campaign to reduce death from vaccine-prevented diseases (VPDs) is in jeopardy, just as COVID-19 caused a comparable breakdown of immunization systems [10].

In low- and middle-income countries (LMICs), it is anticipated that the disruption to routine healthcare services due to the COVID-19 pandemic will result in more maternal and child fatalities [10–12]. Due to several reasons, including the interruption of mass measles routine vaccination, a monopoly focus on COVID-19 eradication, hunger, and inadequate surveillance, the development of COVID-19 has worsened the morbidities and mortality of measles [1, 10, 13]. WHO advised against continuing mass vaccination drives to stop the spread of COVID-19 in communities, which could have an immediate impact on immunization rates, particularly in rural and underserved areas [14]. There has been a recent increase in measles infections in Sub-Saharan Africa, with the number of cases reaching 17,500 as of January 2022, a 400% increase from cases reported in 2021 [15, 16]. Another study showed that to deal with SARS-CoV-2, 23 countries had paused their measles vaccine campaigns, and the world's most contagious virus has already claimed thousands of lives in the Democratic Republic of the Congo [17]. The consequences of these disturbances were especially noticeable in Sub-Saharan Africa, where the healthcare system was already experiencing difficulties prior to the epidemic. The measles and Rubella Initiative reports that low vaccination rates led to serious measles epidemics in countries including Nigeria, Yemen, and the Democratic Republic of the Congo. This also applied to Zambia. Zambia had a measles vaccination coverage rate of about 91% prior to the pandemic, but UNICEF (2021) [18] reports that during the pandemic, this fell to 83%. A rebound of measles cases and higher mortality, especially among children under five, resulted from the immunity gap this reduction produced.

The health system in Zambia was similarly overburdened, which made it difficult to continue providing basic medical services like regular immunization campaigns. Zambia's measles vaccine coverage fell from 91% in 2019 to 83% in 2021, mostly as a result of resources being diverted for the COVID-19 response, according to UNICEF. This decrease was accompanied by a rise in measles epidemic reports [19, 20]. Since measles is a highly contagious disease, herd immunity depends on high vaccination rates of 95% or higher and ooutbreaks can result from very slight reductions in coverage [21]. Missed vaccinations during the pandemic have led to a global comeback of vaccine-preventable diseases, according to research [22]. Although Zambia's predicament mirrors this worldwide pattern, local analysis is necessary to guide context-specific measures.

The COVID-19 pandemic made it more difficult to conduct routine and mass measles vaccination, increasing the number of unvaccinated children who are susceptible to the disease, leading to a double burden of cases and gross fatality rate [23]. However, the reports before COVID-19 pandemic showed an improvement in the measles-containing vaccine (MCV1) coverage in Africa, reaching around 69% by 2019 [24]. There is little information available about how COVID-19 impacted routine vaccinations and outbreak response mechanisms in Zambia, despite regional studies showing a rise in measles outbreaks during the pandemic. In addition, by ensuring that vaccination programs are not deprioritized during future emergencies, the findings may help Zambia prioritize measles control in its health policy and funding strategies. Research emphasizes how crucial it is to continue providing normal vaccination services in times of emergency to stop subsequent epidemics [25, 26]. The results of this study would advance global understanding of practical methods for preserving vaccination coverage during pandemics. Therefore, this study examined the effects of the COVID-19 pandemic on measles mortalities and associated predictors among patients seen at health facilities in Zambia.

## Methods

### Study design

Interrupted Time Series (ITS) design analysis was applied to evaluate the impact of COVID-19 effects on measles mortality. We compared Zambia's monthly measles mortality rates before and after the COVID-19 pandemic using an interrupted time series design. The period running from January 2017 to February 2020 just before the first reported COVID-19 case was defined as pre-COVID-19, and March 2020 to December 2023 as post-COVD-19. These data were deemed appropriate as it was collected at monthly intervals and were consistent with interrupted time series designs [20, 27]. We used this data to determine an overarching trend, and considered COVID-19 as an intervention that took place from March 2020. We thus considered the period after as post-intervention to establish the trends. We then made comparisons of the trends using the pre-existing trends as counterfactual [28]. We reviewed retrospective routine data collected from 2017 to 2022. Zambia detected and reported the first case of COVID 19 in March 2020, and so we arbitrarily used February 2020 as the defining timepoint for pre- and post-COVID 19.

Study area.

The pandemic was a major global health challenge that tested the resilience of health systems around the world. In Zambia, the pandemic shown the resolve of communities and healthcare workers to respond effectively while also exposing critical weaknesses in the health system. The decentralized structure of Zambia's health system allows services to be provided at the local (health facility), district, and provincial levels. To manage public health emergencies, the Zambia National Public Health Institute (ZNPHI) assists the Ministry of Health (MOH), which is in charge of healthcare delivery (World Health Organization [29]. Notwithstanding these frameworks, the pandemic exposed serious weaknesses in readiness. Essential resources including oxygen concentrators, personal protective equipment (PPE), and intensive care units were lacking in many healthcare facilities, particularly in rural areas. These flaws made it more difficult for the system to handle severe COVID-19 cases, especially in isolated locations with inadequate healthcare infrastructure [30]. Routine health services were severely affected by the allocation of resources to the COVID-19 reaction. As caregivers shunned medical facilities out of fear of infection, immunization programs saw a drop [31]. Measles outbreaks and other vaccine-preventable illnesses were caused by this interruption [18, 29]. Reductions in prenatal care and competent birth attendance contributed to poor mother and neonatal outcomes, and maternal and child health services were also impacted. Service delivery disruptions, such as postponed medicine refills and decreased community outreach, also affected Zambia's generally strong HIV and TB programs [32, 33]. These interruptions brought to light how vulnerable vital healthcare services are in times of public health crises [31].

### Case-based surveillance

Any patient in whom a clinician suspected measles was required to fill out a case investigation form (CIF) which was sent to the laboratory along with a serum sample. This was the procedure for all suspected cases meeting the “measles” case definition of febrile rash with at least one of the symptoms, cough, coryza, or conjunctivitis. Urine samples and/or throat swabs were not routinely taken throughout this time.

### Specimen collection

Blood samples of about 2 mls were collected from study participants by phlebotomist and health care workers and using the available hospital courier systems, the collected samples were transported to University Teaching Hospital (UTH) virology laboratory for testing. The testing was conducted in line with the laboratory protocols for measuring measles-specific antibody titres in Zambia. Measles IgG test kits were bought in consultation with the Zambia National Public Health Reference Laboratory. Laboratory quality controls for the test kits were done and were complying. The measles IgG test kits used in this study were OriGene Measles Test Kits [34]. Using a commercial enzyme-linked immunosorbent assay (ELISA), serum samples received at the reference laboratory in Lusaka were examined for measles immunoglobulin M (IgM) and rubella IgM in accordance with manufacturer recommendations. Sera were examined between 2022 and 2023 using Enzygnost® kits from Siemens AG in Erlangen, Germany. Sera were examined using Euroimmun® kits (Euroimmun AG, Luebeck, Germany) during the years 2022–2023. Any ambiguous measles IgM results were followed by a request for a second sample [34, 35].

### Case definition

#### Suspected case

A person who exhibits measles-like symptoms, such as fever, a non-vesicular, widespread maculopapular rash, and at least one of the following symptoms, cough, coryza (runny nose), or conjunctivitis (red eyes), is considered a suspected case. Regardless of a person's age or vaccination history, this classification is consistently utilized to identify possible cases during outbreaks. To confirm or rule out measles in suspected cases, more research is necessary, such as laboratory testing or epidemiological evaluations [21].

#### Probable case

Even in the lack of laboratory confirmation, a suspected case with additional evidence supporting a measles diagnosis is considered a probable case. A significant epidemiological connection to a confirmed measles case, such as close contact with an infected person throughout the incubation period, may be one example. It may also depend on clinical judgment, especially in regions, where measles symptoms are prevalent and outbreaks are continuing. When tracking the spread of measles, probable cases are crucial, particularly in environments with few laboratory resources [21].

#### Confirmed case

A case that satisfies certain requirements for a measles diagnosis is considered confirmed. Polymerase chain reaction (PCR) testing, virus isolation, and positive IgM serology are examples of laboratory confirmation. Even in the absence of laboratory testing, a confirmed case may be established by meeting clinical criteria during a period of extensive transmission or by an epidemiological link to another confirmed case. Accurate public health interventions depend on laboratory confirmation to differentiate measles from other diseases with comparable symptoms, such dengue or rubella [36].

In this study, we only report on the laboratory-confirmed cases and do not further discuss the compatible cases, due to the heterogeneous nature of febrile rash aetiology in years with no measles outbreaks. A vaccinated measles case was defined as a patient who received either first or second or both doses of the measles vaccine administered routinely in Zambia to infants at 9 months of age and toddlers at 18 months. Furthermore, individual patient data were considered if it contained patient status (died or alive), sex, age, whether fever or not, cough or not, red eyes or not, runny nose or not and rash or not) and measles IgM (positive or negative).

### Sample size determination

Previous studies indicate that the proportion of deaths due to measles is 0.028 for unvaccinated and 0.006 for vaccinated [37, 38]. We determined the minimum sample size required to detect an absolute difference with 80% power using 5% level of significance with odds ratio of dying at 4.7 odds [21]. The computed minimum sample size for this study was 1, 082 participants using this formula accounting for none response and cluster effect:

$${\text{n}} = \frac{{\left[ {{\text{Z}}_{1 - \alpha /2} \sqrt 2 {\text{P}}^{ - } {\text{Q}}^{ - } - {\text{Z}}_{1 - \beta } \sqrt {\text{P}}_{1} {\text{Q}}_{1} + {\text{P}}_{2} {\text{Q}}_{2} } \right]^{2} }}{{({\text{P}}_{{2}} {-}{\text{P}}_{{1}} )^{{2}} }}$$

### Data sources

We used the District Health Information Software 2 (DHIS2) for the Health Management Information System (HMIS) to access the national data that is gathered from all healthcare facilities. Monthly aggregated facility level data is entered into the system by health records and information officers at different health institutions using a variety of devices, such as desktop computers, laptops, tablets, and cell phones. A central administrative entity receives the data, pools it, and enters it into the national database. The health facility staff aggregates the data using a standard paper-based service delivery aggregation form 2 (HIA2) on a monthly basis. After verifying the information, the District Health Office (DHO) inputs it into a nationwide online DHIS2 portal [22] A monthly average of 80% is the acceptable report completeness percentage for vaccinations and cases. The data indicates that the completeness threshold was exceeded each month, with an average complete report rate of 94.0% for the observation period. Over time, even during the pandemic, the completeness increased (see Table 1). In addition, we divided the projected population for each year by 12 months to account for the denominators and estimate coverage, which varied from year to year due to population growth, using static population estimates from the 2010 projected census population estimates provided by the Zambia Statistics Agency (ZamStats) [39]. Each month had the same denominator. According to other reports, vaccination coverage was defined as the proportion of age-eligible children who received their vaccinations by 30 or 31 days after the due date (28 or 29 days after the February due dates). Since the population is not expected to fluctuate from month to month but rather on an annual basis, the population is calculated by dividing the annual anticipated population by the number of months. We also regularly give and record our data, thus we relied on the information provided by the system for each vaccine [39]. The acceptable report completeness percentage for immunization is an average of 80% every month. The data reveals every month it was over the criterion for completeness and the mean value was 94.0% of complete report rate for the period of observation [33]. Over time, even during the pandemic, the completeness increased (see Table 2).

**Table 1:** Routine childhood vaccination calendar in Zambia

Vaccine	Age of administration	Purpose/target disease	Doses required
Bacillus Calmette–Guérin (BCG)	At birth	Tuberculosis	1
Oral Polio Vaccine (OPV)	At birth, 6 weeks, 10 weeks, 14 weeks	Polio	4
Pentavalent Vaccine (DTP-HepB-Hib)	6 weeks, 10 weeks, 14 weeks	Diphtheria, Pertussis, Tetanus, Hepatitis B, Haemophilus influenzae type b	3
Measles-Containing Vaccine (MCV1)	9 months	Measles	1
Measles-Containing Vaccine (MCV2)	18 months	Measles	1
Rotavirus Vaccine	6 weeks, 10 weeks	Severe diarrhea caused by rotavirus	2
Pneumococcal Conjugate Vaccine (PCV)	6 weeks, 10 weeks, 14 weeks	Pneumonia, Meningitis caused by pneumococcus	3
Human Papillomavirus (HPV) Vaccine	9 years (for girls)	Cervical cancer	2

**Table 2:**
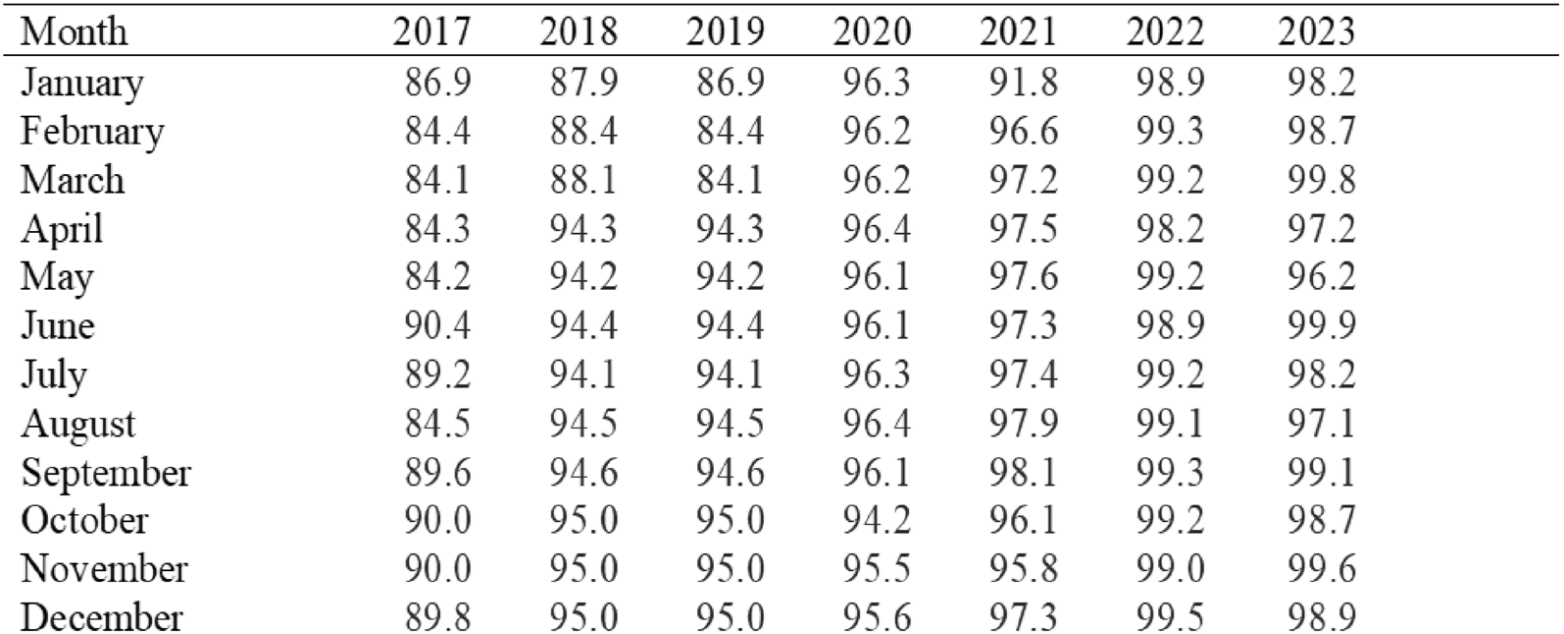
Data completeness from 2017 to 2023 by month

Furthermore, the facility-based immunization registers, notifiable diseases form for patient level data (ND-1) were used to capture patient information. The measles suspected case line list form was used to gather information from all cases that mate the clinical case description. To get the data for the questionnaire, parents were questioned on their children's immunization history. Additional data was gathered from paper or electronic medical records. By matching the patient IDs, duplicates and unidentified patients were found and eliminated. Included were the date of admission, the place of exposure as reported by the caregiver, the diagnosis at the time of admission, the measles vaccination status as shown on the vaccination card (if available), sociodemographic information, clinical characteristics, and the outcome. The characteristics of the rash, child’s age, sex of the patient, vaccinated status, fever, red eyes, running nose, cough and measles IGM status.

### Data analysis

The characteristics of the patients were compiled using descriptive statistics. Frequencies and percentages were used to display categorical variables. The chi-squared test of independence was used to look for statistically significant correlations between categorical variables.

Univariable logistic regression was applied to determine the factors that predicted mortality among the measles patients. Multiple logistic regression was used to identify predictors of mortality using an investigator-led stepwise regression procedure. The variables for the final model were chosen by first running the multiple logistic regression command with all of the predictor variables. Next, predictor variables with the highest *p* values from the model were removed one after the other until only those that best predicted the outcome remained in the model. The multivariable analysis model contains four explanatory variables; child’s age, sex of the patient, vaccinated status, and measles IGM as the best predictors of health mortality among measles patients. Although patients’ age was not statistically significant, the variables was left in the model due to priori knowledge from other studies which consistently showed that they could be used to perfectly predict mortality. Finally, based on the Akaike's Information Criterion (AIC) and Bayesian Information Criterion (BIC) for the competing models, the best fit model was chosen. The model that had the lowest AIC and BIC values in comparison with other models was picked. The 95% confidence intervals (CI) for the crude odds ratio (cOR) and adjusted odds ratios (aOR) were generated. A *p* value of less than 0,05 was regarded as significant.

To examine mortality trends in both periods (pre- and post-COVID-19) and estimate the effect size (change in slope due to COVID-19), we employed segmented quasi-Poisson regression analysis. We included an offset variable (population growth over the years) to transform the result into a rate; and to account for population fluctuations over time we age-standardized our sample in person-years given the relatively instability over time [40]. To account for seasonal impacts that could create consistent highs and lows in data we used seasonal models with harmonic terms that take seasonal factors into account [28, 41, 42]. While there are various methods of adjusting for seasonality, we used harmonic terms specifying the number of sine and cosine pairs to include (in this case 2) and the length of the period (12 months) as reported elsewhere [40]. The time sequencing of data points used in time series analysis can cause residual autocorrelation to violate regression assumptions. To produce more conservative estimates of uncertainty, robust standard errors were generated (using a sandwich estimator) in cases where significant residual autocorrelation was detected (P < 0.10) and the assumptions of the general linear models became problematic [43]. R statistical software (version 3.3.2; RStudio, Inc.) was used for all analyses (version 1.0.136; RStudio, Inc) and STATA 16 (STATA Corp.) was used for the analysis.

The equation that wase used in this analysis was

$$\begin{aligned} {\text{Yt }} = & {\text{b}}0 \, + {\text{ b1T }} + {\text{ b2Xt }} + {\text{ b3TXt }} + {\text{ b4sin}}\left( {\left( {{\text{2pi}}/{12}} \right)*{\text{m}}} \right) \, + {\text{ b5cos}}\left( {\left( {{\text{2pi}}/{12}} \right)*{\text{m}}} \right) \, + {\text{ b6sin}}\left( {\left( {{\text{4pi}}/{12}} \right)*{\text{m}}} \right) \, \\ & + {\text{ b7cos}}\left( {\left( {{\text{4pi}}/{12}} \right)*{\text{m}}} \right) \\ \end{aligned}$$

where b0 represents the baseline level at T = 0, b1 is the change in outcome associated with a time unit increase (representing the underlying pre-COVD-19 trend), b2 is the level of change following the COVID-19 period, b3 indicates the slope change following the COVID-19 (using the interaction between time and COVID-19: TXt), m is the calendar month, and b4–b7 are coefficients on the seasonal adjustment terms.

### Ethical considerations

This work involved secondary analysis with no individually identifiable data. Authorization to conduct the study was sought from the University of Zambia Ethical Research Committee (UNZABREC) number REF. No. 5434-2024.

## Results

During the period under review, 3429 measles cases were reported. Of these, only 1261 patients had complete hospital-line lists information and were considered for analysis.

### Study population characteristics and overall patient outcome frequency

**Fig. 1:**
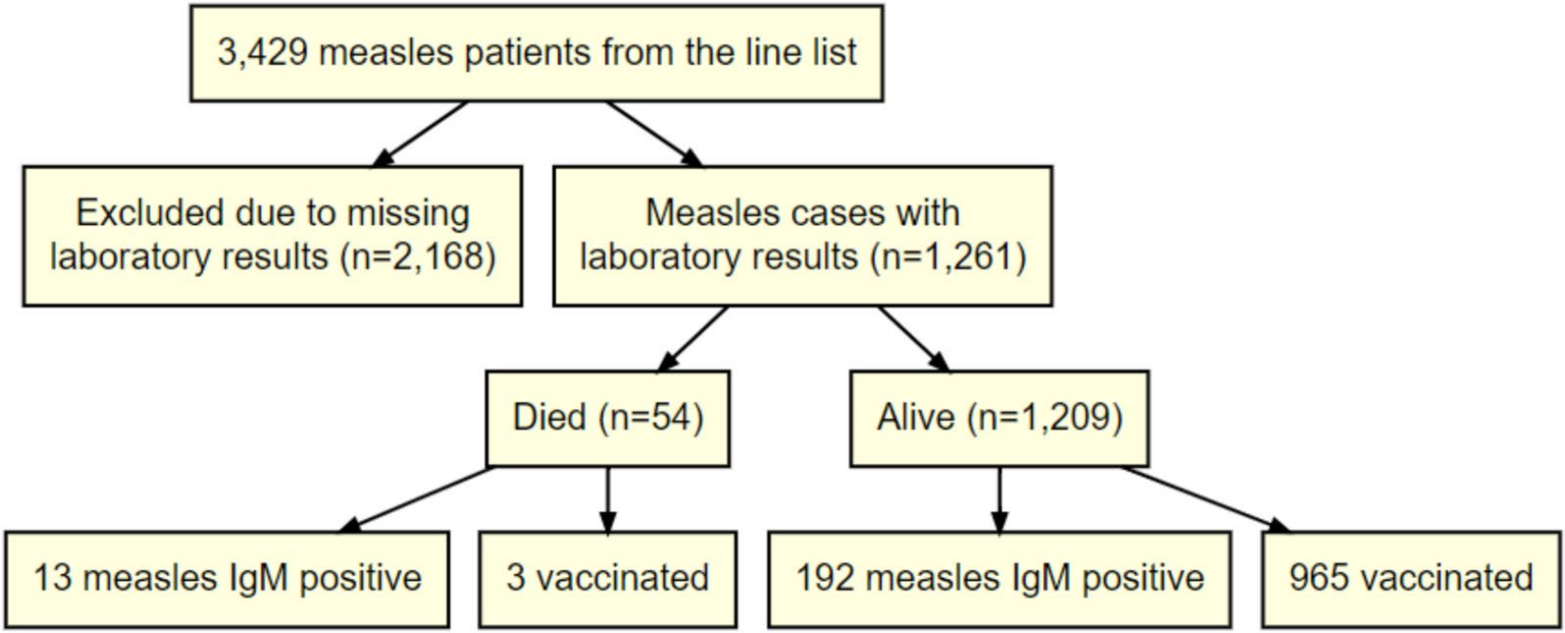
Figure highlights the distribution and outcomes of the confirmed measles cases, emphasizing differences in vaccination and IgM positivity status between those who survived and those who died

The median age of the patients was 3 years (IQR, 1–7) with a higher proportion of female 52.9%. Fifty-four (4.3%) patients died, while 1207 (95.7%) did not die. (Case fatality rate was 4.47) as shown in Fig. 1. Out of the sample analysed, the majority (79.0%) had a negative measles IgM, while 205 (21.0%) were positive. Most of the patients (83.1%) were vaccinated. Majority had either a fever (89.0%), a cough (94.4%), red eyes (92.3%), a runny nose (89.4%), or a rush (22.0%) (Table 3). (Whether fever or not, cough or not, red eyes or not, runny nose or not and rash or not) there was evidence of a difference evidenced by *p* value greater than 0.05. However, there was a statistical difference in age group, sex, and vaccination status between those who knew whether the patient died or did not die, evidenced by the p-value being value less than 0.05 (Table 3).

**Table 3:** Demographic and clinical characteristics of measles patients

Patient characteristics	Measles patients (*N *= 1259)	Died (*N* = 54, 71.1%)	Alive (*N* = 1209, 95.7%)	*p* value
	*N* (% of total)	*N* (%)	*N* (%)	
Age group				
0–4 years	736 (60.1)	35 (72.9)	701 (59.6)	
5–9 years	314 (25.6)	6 (12.5)	308 (26.2)	
10–15 years	108 (8.8)	2 (4.2)	106 (9.0)	
Over 15 years	66 (5.4)	5 (10.4)	61 (5.2)	0.032^C^
Sex				
Female	594 (47.1)	33 (61.1)	561 (46.5)	
Male	667 (52.9)	21 (38.9)	646 (53.5)	0.035^C^
Measles IGM				
Positive	205 (21.0)	13 (24.1)	192 (20.8)	
Negative	770 (79.0)	41 (75.9)	729 (79.2)	0.572^C^
Vaccinated				
Yes	207 (16.9)	3 (5.6)	965 (82.6)	
No	1016 (83.1)	51 (94.4)	204 (17.4)	0.023^C^
Fever				
Yes	1122 (89.0)	1071 (88.7)	51 (94.4)	
No	139 (11.0)	136 (11.3)	3 (5.6)	0.190^C^
Cough/Coryza				
Yes	1187 (94.4)	1136 (94.4)	51 (94.4)	
No	70 (5.6)	67 (5.6.5)	3 (5.6)	1.000^E^
Red eyes				
Yes	1123 (92.3)	49 (94.4)	1074 (92.3)	
No	94 (7.7)	4 (7.6)	90 (7.7)	1.000^E^
Runny nose				
Yes	1072 (89.4)	50 (92.6)	1022 (89.3)	
No	127 (10.6)	4 (7.4%)	123 (10.7)	0.649^C^
Rash				
Yes	857 (22.0)	42 (89.4)	815 (77.6)	
No	241 (857)	5 (10.6)	236 (22.4)	0.056^C^

P: *Z* test of two proportions; C: Chi squared test; E: Fisher’s exact test; W: Wilcoxon rank sum test; N: number of participants; IgM: Immunoglobulin M; %: Percentage

### Predictors of mortalities among measles patients

A multivariable logistic regression was performed to examine the predictors of mortalities among measles patients. The results of univariate analysis, that is, crude odds ratios (cOR) in Table 4 showed that patients, that is, who were vaccinated were 0.28 times the odds of dying compared to patients who were not vaccinated (cOR = 0.28; 95% CI 0.09–0.90). Whether the patient was male or female, patients who were female had 1.81 times the odds of dying compared to male patients (cOR = 1.81; 95% CI 1.03–3.16) and patient aged 0–4 years were more likely to die (cOR = 2.56; 95% CI 1.06, 6.25) taking the reciprocal of odd ratio. On the other hand, measles IGM (cOR = 1.20; 95% CI 0.63–2.29), Fever (cOR = 2.16; 95% CI 0.66–7.01), Cough (cOR = 1.00; 95% CI 0.30–3.30), Red eyes (cOR = 1.03; 95% CI 0.36–2.91), Runny nose (cOR = 1.50; 95% CI 0.53–4.24) and Rash (cOR = 2.43; 95% CI 0. 0.95–6.22) had no statistically significant association with mortality.

**Table 4:** Logistic regression crude and adjusted odd ratios of measles patients

Factors	Crude Odd Ratio (95% CI)	*p* value	Adjusted Odd Ratio (95%CI)	*p* value
Age, years				
0–4 years	Ref (1)		Ref (1)	
5–9 years	0.39 (0.16, 0.94)	0.035	0.40 (0.16, 0.97)	0.042
10–15 years	0.38 (0.10, 1.60)	0.185	0.13 (0.02, 1.25)	0.078
Over 15 years	1.64 (0.62, 4.34)	0.318	1.61 (0.50, 5.20)	0.466
Sex				
Male	Ref (1)		Ref (1)	
Female	1.81 (1.03, 3.16)	0.037	1.84 (0.94, 3.59)	0.089
Measles IGM				
No	Ref (1)		Ref (1)	
Yes	1.20 (0.63, 2.29)	0.187	3.97 (1.71, 6.78)	0.007
Vaccinated				
No	Ref (1)		Ref (1)	
Yes	0.28 (0.09, 0.90)	0.033	0.07 (0.01, 0.59)	0.023
Fever				
No	Ref (1)		Ref (1)	
Yes	2.16 (0.66, 7.01)	0.200	2.50 (0.32, 13.10)	0.246
Cough				
No	Ref (1)		Ref (1)	
Yes	1.00 (0.30, 3.30)	0.997	0.65 (0.10, 4.17)	0.565
Red eyes				
No	Ref (1)	Ref	Ref (1)	
Yes	1.03 (0.36, 2.91)	0.961	0.34 (0.06, 1.94)	0.220
Runny Nose				
No	Ref (1)		Ref (1)	
Yes	1.50 (0.53, 4.24)	0.440	2.94 (0.13, 3.06)	0.480
Rash				
No	Ref (1)		Ref (1)	
Yes	2.43 (0.95, 6.22)	0.063	2.94 (0.85, 9.01)	0.123

As shown in Table 5, age, sex, measles status, vaccination status, and the presence of rash were significantly associated with mortality. The following predictors were identified: younger patients in the 0–4 year age category had significantly higher odds of mortality (AOR = 2.78; 95% CI 1.16–7.14). In contrast, age groups 10–15 years and over 15 years showed no statistically significant association with mortality, controlling for other predictors in the model. Vaccinated patients had significantly reduced odds of dying, with a 94% reduction in mortality risk compared to unvaccinated patients (AOR = 0.06; 95% CI 0.01–0.46). Patients with a positive measles IgM result were 2.17 times more likely to die compared to those with a negative result, after adjusting for other predictors (AOR = 2.17; 95% CI 1.07–4.39). Female patients were found to have higher odds of mortality compared to males (AOR = 1.90; 95% CI 1.04–3.50). Patients who presented with a rash were 3.7 times more likely to die compared to those without rash (AOR = 3.66; 95% CI 1.31–6.21).

**Table 5:** Multivariable analysis

Measles mortality	Odds ratios	95% Confidence intervals	*p* values
Age, years			
0–4 years	Ref	(1)	
5–9 years	0.36	(0.14, 0.86)	0.023
10–15 years	0.28	(0.06, 1.23)	0.091
Over 15 years	1.37	(0.49, 3.80)	0.550
Sex			
Male	Ref	(1)	
Female	1.90	(1.04, 3.50)	0.039
Measles IGM			
No	Ref	(1)	
Yes	2.17	(1.07, 4.39)	0.007
Vaccinated			
No			
Yes	Ref	(1)	0.005
Rush			
No	Ref	(1)	
Yes	3.66	(1.31, 6.21)	0.013

### Effect of COVID-19 pandemic on trend of reported measles mortality

**Fig. 2:**
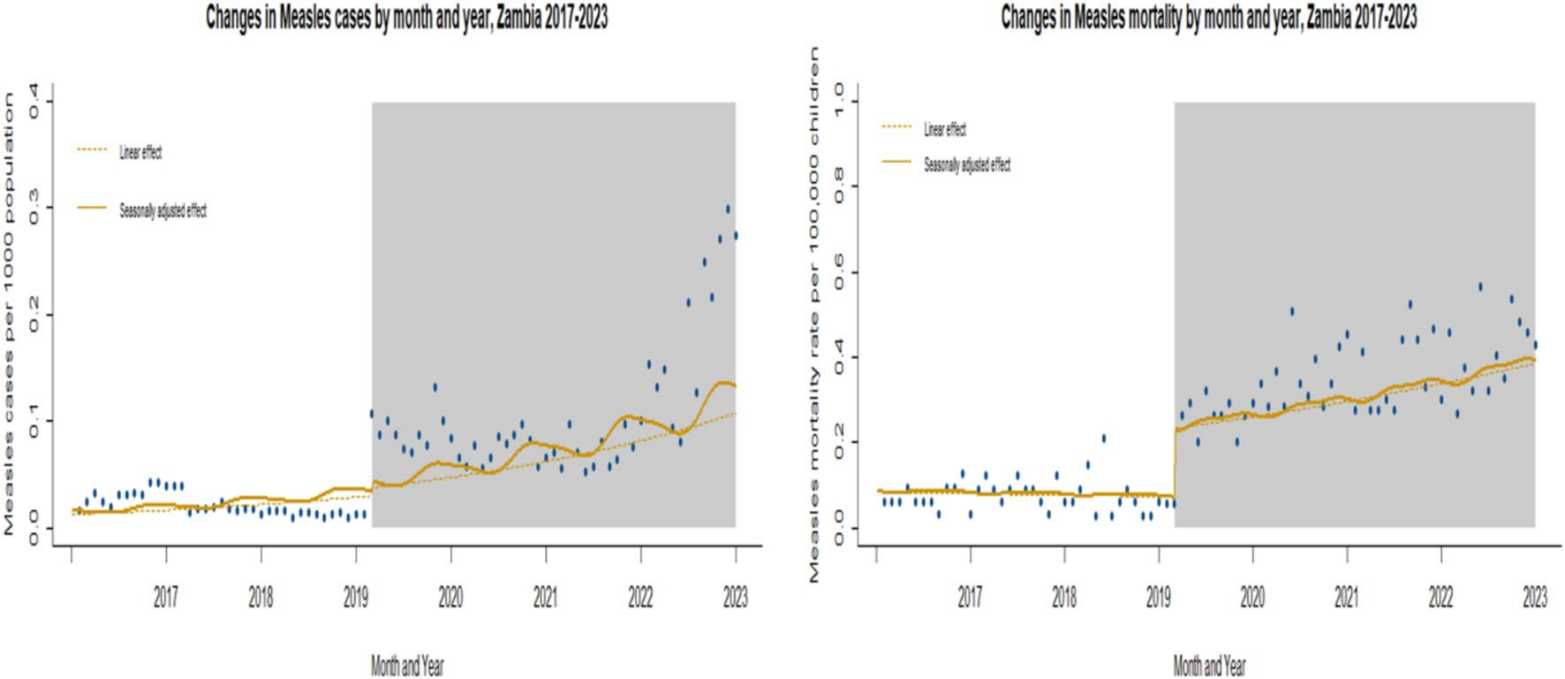
Trends in measles mortality and cases pre and post the onset of the COVID-19 pandemic in Zambia (2017–2023). Data points represent monthly measles case and mortality rates from 2017 to 2023. The gray-shaded area highlights the COVID-19 pandemic period in Zambia. Dashed lines indicate fitted estimates using a segmented linear step-change model, while the curved lines represent seasonally adjusted fitted values. The figure illustrates the significant rise in measles cases and mortality during the pandemic compared to pre-pandemic trends, accounting for both seasonal patterns and overall changes

**Fig. 3:**
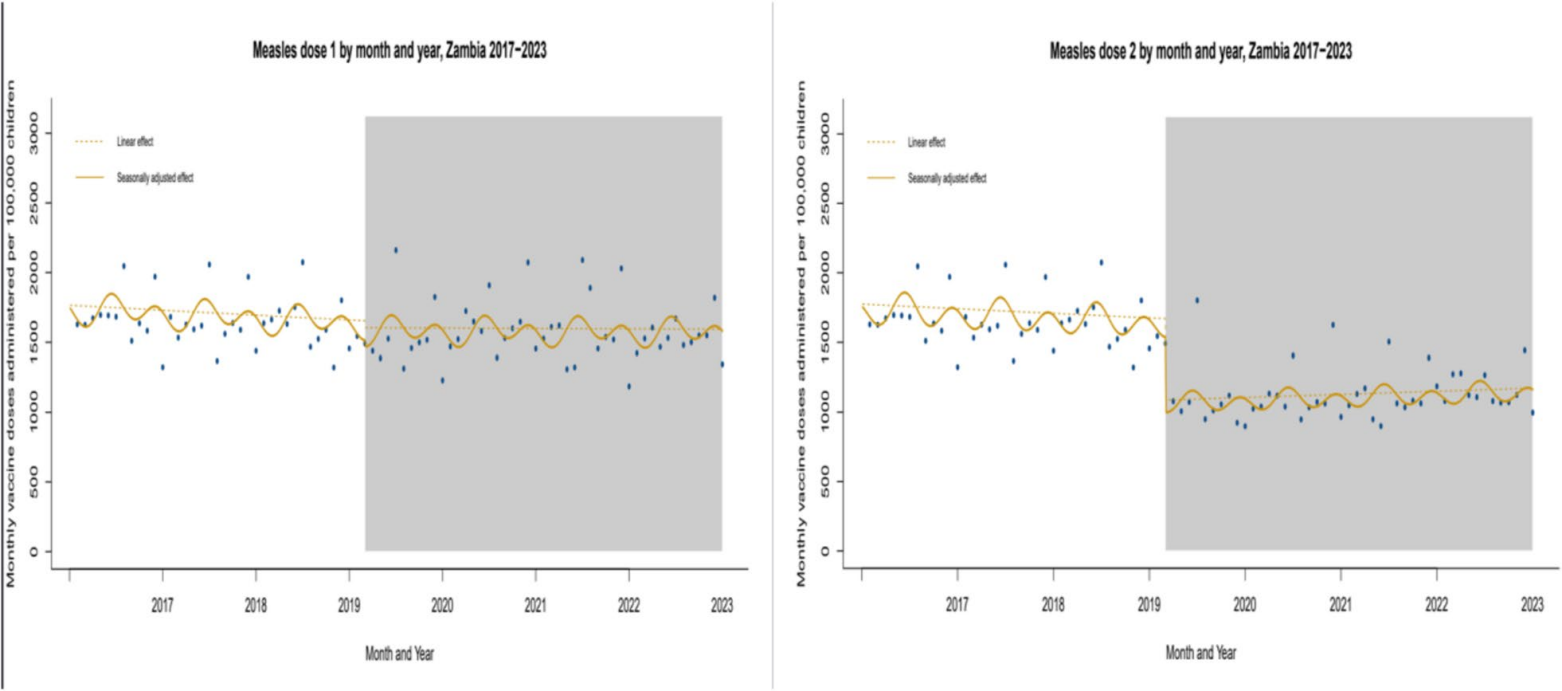
Trends in measles vaccine coverage (MCV1 and MCV2) pre and post the COVID-19 pandemic in Zambia (2017–2023). Data points represent monthly vaccination rates for MCV1 (Measles Containing Vaccine Dose 1) and MCV2 (Dose 2) from 2017 to 2023. The gray-shaded area highlights the COVID-19 pandemic period in Zambia. Dashed lines show fitted estimates using a linear step-change model, while the curved lines represent seasonally adjusted fitted values. The figure illustrates the decline in MCV2 coverage during the pandemic, with no significant disruption observed for MCV1

Between January 2017 and February 2020 (pre-COVID-19 period), Zambia recorded a mean monthly measles mortality count of 2, corresponding to a mortality rate of 0.1 deaths per 100,000 population children as shown in Fig. 2. In contrast, post COVID-19 pandemic period (March 2020 to December 2023), the mean monthly mortality count increased to 12, with a mortality rate of 0.22 deaths per 100,000 population children (Table 4). After adjusting for underlying trends, we estimated a 2% increase in the monthly measles mortality trend during the post COVID-19 pandemic compared to the pre-COVID-19 period (Rate Ratio [RR] = 1.02; 95% CI 1.00–1.04; *p* = 0.017) as shown in Table 6. In addition, a significant trend change in mortality was observed, with an 81% increase in the rate ratios of the change (RR = 1.81; 95% CI 1.14–2.87). The trend of measles case counts also increased significantly at the last 2 years of the post pandemic period (RR = 1.04; 95% CI 1.01–1.06). The COVID-19 pandemic had a notable impact on vaccination coverage. Coverage for the second dose of the Measles-Containing Vaccine (MCV2) was the most affected, with monthly figures dropping from 52,820 to 40,418 doses per 100,000 population children immediately following the onset of the pandemic as shown in Fig. 3. Interestingly, coverage for the first dose (MCV1) remained relatively stable throughout the pandemic period under study. Trends in monthly MCV2 vaccinations later increased slightly during the post pandemic period, with a growth rate ratio of 0.3% (RR = 1.003; 95% CI 1.001–1.005). However, there was a sharp decline in MCV2 coverage immediately after the pandemic's onset, with a 42% reduction (RR = 0.58; 95% CI 0.46–0.72).

**Table 6:** Segmented regression analysis of relative risk (RR) for measles-containing vaccine coverage (MCV1 and MCV2), measles cases, and mortality rates pre- and post-COVID-19 pandemic

Type of vaccine	Mean monthly count		Mean monthly rate per 100,000		Slope change Pre-COVID-19	*p* value	Slope change during-COVID-19	*p* value	Step change	*p* value
	Before COVID-19	After COVID-19	Before COVID-19	After COVID-19	RR (95% CI)^a^		RR (95%CI)^C^		RR (95%CI)^c^	
Measles cases	78.1	387.6	0.02	0.11	0.99 (0.97–1.01)	0.398	1.04 (1.01–1.06)	0.004	0.67 (0.31–1.45)	0.3034
Measles mortality	1.9	8.7	0.1	0.22	0.99 (0.98–1.01)	0.558	1.02 (1.00–1.03)	0.028	1.81 (1.14–2.87)	0.011
Measles dose 1	53,465	56,493	1643	1575	0.998 (0.994–1.001)	0.063	1.00 (0.99–1.01)	0.240	0.91 (0.78–1.07)	0.271
Measles dose 2	52,820	40,418	1628	1126.4	0.998 (0.996–1.000)	0.063	1.003 (1000–1.010)	0.042	0.58 (0.46–0.72)	0.0001

Data points represent monthly measles case and mortality rates from 2017 to 2023. The grey-shaded area highlights the COVID-19 pandemic period in Zambia. Dashed lines indicate fitted estimates using a segmented linear step-change model, while the curved lines represent seasonally adjusted fitted values. The figure illustrates the significant rise in measles cases and mortality during the pandemic compared to pre-pandemic trends, accounting for both seasonal patterns and overall changes.

Data points represent monthly vaccination rates for MCV1 (measles containing vaccine dose 1) and MCV2 (dose 2) from 2017 to 2023. The grey-shaded area highlights the COVID-19 pandemic period in Zambia. Dashed lines show fitted estimates using a linear step-change model, while the curved lines represent seasonally adjusted fitted values. The figure illustrates the decline in MCV2 coverage during the pandemic, with no significant disruption observed for MCV1.

## Discussion

Measles vaccine coverage and mortality patterns in Zambia were severely affected by the COVID-19 pandemic. Following the start of the pandemic, MCV2 coverage fell precipitously by forty 2%, indicating disruptions in regular healthcare services. MCV1 coverage, on the other hand, showed resilience by remaining steady over the course of the period. With a higher mean monthly count and mortality rate in the post-pandemic period than in the pre-pandemic years, measles mortality also rose. Significant changes in disease dynamics during the pandemic were indicated by an 81% trend change and a 2% monthly rise in the death trend. The study also found that female sex, younger age less than five, testing positive for measles IgM, and having a rash were important predictors of mortality among measles patients. Significantly increased probabilities of death were linked to these characteristics. On the other hand, vaccination was discovered to be a powerful protective factor, significantly lowering the risk of passing away.

The finding of this study shows that children below 5 years experienced high mortality compared to those aged 5 years and above. This finding is consistent with other studies that determined measles mortalities among age groups [28, 29, 44]. However, following the age of four, there were no appreciable differences between the actual vaccine coverage rates (observed values) and the projected rates based on historical trends [45]. Around 80 million children under 1 year have been impacted by the COVID-19 pandemic's disruption of childhood immunization systems in at least 68 countries in 2020 [1, 27, 44]. Another study revealed that the case fatality ratio was highest in children under the age of five and ranged from 2.8 to 7% [46]. This happened for a number of reasons, including the diverting of medical personnel, resources, and funds to deal with COVID-19 treatment and response; people's reluctance to bring children to be vaccinated due to fear of infection; travel restrictions due to local physical distance measures; disruptions in vaccine supply chains; a lack of personal protective equipment; and decisions to halt or postpone vaccination campaigns to reduce the risk of transmission during such events [2, 28, 29]. Moreover, routine immunization coverage in Zambia frequently falls below the 95% threshold recommended by the World Health Organization (WHO) for herd immunity; for example, WHO data show that Zambia's second-dose measles vaccine (MCV2) coverage was only 85% in 2022, leaving many children unprotected and at risk of infection [18, 43]. Research indicates that malnourished children, especially those with vitamin A deficiency, are at greater risk of severe complications from measles, including pneumonia, encephalitis, and blindness [47]. According to another study suggests that the COVID-19 pandemic has further disrupted routine immunization services, widening immunity gaps and increasing the risk of outbreaks in children [48]. Furthermore, recurrent measles outbreaks strain Zambia's healthcare system and impede progress toward regional measles elimination goals. The cross-border nature of outbreaks in Southern Africa necessitates collaborative regional approaches to strengthen surveillance, harmonize immunization schedules, and enhance cross-border health interventions. Developing and maintaining at least 95% coverage for the first and second doses of the measles-containing vaccine (MCV1 and MCV2) is crucial to preventing outbreaks, and Zambia must take a multifaceted approach to addressing the burden of measles infections among children under five. This can be done by expanding outreach to underserved and rural communities and integrating immunization services into larger child health programs, such as nutrition and growth monitoring initiatives [49].

A previous study that assessed mortality due to measles found an excessive amount of female death has been reported in South Asia [50, 51]. Our finding has also shown that females are more likely to die than males. The finding which are closer to separate studies done in Nigerian and Senegal that looked at mortality rates due to sex difference and their variations of exposures [52–55]. In contrast to our finding, our current findings are different from a report that found that in all ages, females generally have a lower mortality rate than men [45]. In a separate study, it is reported that biological variations may be one factor contributing to the greater measles fatality rate among females. According to another study, women frequently have stronger immune responses than men, which ironically can make some infectious diseases, including measles, more severe [56]. This increased immune response may contribute to greater mortality rates in females by causing excessive inflammation and problems after severe illnesses. Furthermore, health outcomes may be influenced by cultural and societal norms in Zambia and many other sub-regional areas. Due to gendered disparities in decision-making, financial resources, or mobility, women may have less access to healthcare services, including vaccinations, in patriarchal settings [57]. These differences are further exacerbated by socioeconomic variables. Women are more likely to face educational obstacles, especially in rural Zambia, which has an immediate effect on their health literacy and capacity to demand prompt medical attention. As caretakers, they can put their children and other family members'needs ahead of their own, which could cause them to put off getting treatment for severe measles. Delays like this can lead to avoidable consequences, such as encephalitis or pneumonia, which are the main causes of measles-related deaths. In addition, during immunization programs with limited resources, families may prioritize vaccinating male children due to cultural stigmas and gender norms [58]. These findings have significant ramifications for Zambia and the subregion. In addition to impeding the achievement of regional and national health objectives, persistent gender differences in measles mortality also sustain gaps in health outcomes. This gendered burden emphasizes the necessity of focused approaches to resolve systemic injustices and guarantee fair access to healthcare and vaccination services. Reducing unnecessary mortality among females requires a multifaceted strategy. First, priority should be given to gender-sensitive immunization initiatives. Campaigns for vaccination must specifically target female communities, guaranteeing fair access and removing cultural barriers that prevent vaccination uptake. Gender norms can be changed and outreach to vulnerable populations can be improved by community-based initiatives, such as including female health workers and community leaders.

The study has also revealed that the case fatality rate was high (4.47%). The observed CFR for measles during the study period in Zambia was significantly higher, however, within the global average report by the WHO, which is estimated to range between 0.1 and 5% depending on vaccination coverage and access to healthcare services [21]. This discrepancy can be attributed to several contextual factors, including the disruption of herd immunity caused by low vaccination rates during the COVID-19 pandemic, which left many children at risk for severe measles complications, such as pneumonia, diarrhea, and encephalitis, which are the leading causes of infection-related deaths. Zambia's measles CFR is consistent with regional patterns in low-vaccination areas when compared to other sub-Saharan African nations [5, 59]. Studies from nations such as Nigeria and the Democratic Republic of the Congo, for example, have similarly documented significant CFRs during outbreaks, highlighting the urgent need for strong vaccination campaigns in the area [60]. The increased CFR in Zambia during this time, however, raises the possibility that the COVID-19 pandemic's combined impacts further taxed the healthcare system and interfered with regular vaccination campaigns. To lower the CFR in future crises, several strategies need to be used. First, sustaining high coverage rates and averting epidemics require bolstering routine immunization services. The incorporation of immunization services into pandemic preparedness strategies and focused vaccination efforts in underprivileged communities are examples of this [61]. The second way to drastically lower measles-related mortality is to improve healthcare infrastructure and guarantee the availability of necessary supplies, such as vitamin A supplements and medications for subsequent illnesses. Third, there should be a greater emphasis on public health education initiatives to increase knowledge of the significance of timely vaccination and treatment of measles symptoms.

The study findings also show that a positive IgM result was a predictor of mortality. A strong association between a positive IgM result and death found in this study suggests the importance of developing defensive immune system especially in children below 5 years. Various researches have revealed associations between IGM positive and mortality [62–64]. In Zambia, the identification of measles-positive IgM has important ramifications for both public health and the populace's overall well-being. IgM antibodies are a crucial indicator of a recent or acute infection with measles, a highly dangerous viral disease. This finding emphasizes the urgent need for more robust measures to prevent measles outbreaks and lessen their impact in Zambia, where health systems have been disrupted by several issues, including the COVID-19 pandemic. Measles-positive IgM findings indicate active transmission, which can have devastating consequences, especially for vulnerable populations, such as children under 5 years, pregnant women, and immunocompromised individuals. Studies show that in low- and middle-income countries, including Zambia, measles infection is a leading cause of vaccine-preventable deaths among children[65]. Beyond immediate health outcomes, measles infections contribute to long-term immunosuppression, increasing susceptibility to other infections, such as pneumonia and diarrheal diseases [66]. These secondary infections further strain Zambia’s healthcare resources and increase mortality risks, particularly in areas with limited access to healthcare. Reaching health and development objectives is seriously jeopardized by the persistent measles epidemic in Zambia. Outbreaks of measles interfere with the regular provision of healthcare by directing resources into emergency responses and undermining immunization campaigns against other diseases that can be prevented. For instance, the COVID-19 pandemic's reallocation of health resources resulted in lower vaccination rates, which increased the likelihood of outbreaks [67]. Undernourished children are disproportionately affected by measles infections in Zambia, where malnutrition is still a public health concern. This increases the burden of disease and raises the risk of serious consequences and death. Community health workers should be empowered to educate families about the benefits of immunization and dispel myths related to vaccines. Culturally sensitive approaches that involve local leaders and religious organizations can improve vaccine uptake in hesitant communities. It is imperative to raise public awareness about the importance of vaccination and early healthcare seeking behaviour [68]. The severity of measles infections can be decreased by lowering malnutrition and enhancing general health resilience. Immune systems will be strengthened and measles complications will be decreased by incorporating nutritional support, such as vitamin A supplements, into standard child health services.

The study found that vaccinated children had more than 90% chance (94%) of not dying. This highlights the importance of supplemental immunization efforts. This demonstrate that children who are partially or fully immunized could survive mortality due to measles. This study could be compared with another study that found most fatalities were among unvaccinated youngsters and happened at home [28, 32, 33]. The study suggests that low rates of routine measles dose two vaccination, a lack of supplemental immunization efforts, limited access to healthcare, and slow response times to outbreaks all could contribute to an epidemiological environment that encouraged the rapid spread of the measles virus and high rates of morbidity and mortality. National MCV coverage appears to be below the 95% level needed for herd immunity, according to recent statistics [69, 70]. Some LMICs have seen similar patterns. According to a study conducted in Kenya and Nigeria, coverage rates range from 80 to 85%, meaning that a sizable section of the populace is not covered [71]. High-income nations such as the US and Germany, in contrast, have managed to maintain higher vaccination rates; yet, vaccine hesitancy still presents difficulties in these settings [72, 73]. Disparities in healthcare access, vaccine supply chains, and public health messages are frequently the cause of regional variations in vaccination coverage. For instance, delivering vaccines in Zambia's rural and neglected areas presents logistical obstacles, while vaccine reluctance in urban areas may be caused by false information [67, 74]. Designing specialized treatments to increase coverage requires an understanding of these aspects. Due to the diversion of resources to meet the immediate crisis, the COVID-19 pandemic brought to light weaknesses in immunization programs. Nonetheless, pandemics also present a chance to improve and develop vaccination campaigns. Strategies to maximize routine vaccination during pandemics and outbreaks are listed below:

Furthermore, the study findings showed that there was a growing and sloping change in the monthly mortality rate of measles per month after the devastating consequences of the COVID-19 pandemic on the provision of healthcare services. Our review of 72 month worth of data from the health Management Information System (HMIS) revealed evidence of COVID-19's effects on the increase in measles mortality during the pandemic period. Many studies have shown that utilization of health services such as measles vaccination may have gone down due to inadequate supply and underutilization of the health system [59, 75, 76]. Fighting the COVID-19 pandemic has received a lot of attention, and it has had a significant impact on priorities especially child vaccination. [77–80]. Immunization of children has been crucial in lowering the mortality rate for children under five and raising life expectancy in low- and middle-income nations like Zambia. Ensuring access to immunization services and addressing missed vaccination in Zambia, must consider the additional cost of catch-up vaccination for children who have missed doses. Future outbreak responses and preparedness policies must ensure that necessary routine health services, including child immunization services, remain robust during infectious disease outbreaks. A key component of preparedness is the creation of comprehensive immunization plans that are adapted to emergency situations. These plans should include strategies for identifying alternative vaccine delivery methods, prioritizing populations most at risk, and resolving logistical issues. For example, during outbreaks, mobile clinics could be used to make sure vaccines reach underserved and remote areas. These strategies are in line with international guidelines, which emphasize the integration of immunization into larger emergency response frameworks to ensure service continuity [71, 81]. In addition, mmaintaining sufficient vaccination supplies and cold chain infrastructure is another essential component of readiness. Vaccine storage in isolated areas with little access to electricity can be improved by investing in mobile cold storage units and solar-powered refrigeration. These developments are especially pertinent to Zambia, where issues with rural infrastructure can make it difficult to provide vaccines [67]. To avoid interruptions, effective implementation strategies are equally important. For example, decentralizing vaccination delivery can improve access and alleviate congestion at central health facilities. Addressing vaccine hesitancy and dispelling myths during outbreaks requires enlisting the help of local leaders and communities to spread information about the value of routine vaccinations. In comparable contexts, it has been demonstrated that customized communication tactics that align with social and cultural norms boost vaccination rates [71].

The study has important ramifications for public health and policy formation, especially when it comes to enhancing vaccination campaigns and lessening the effects of infectious diseases like measles in Zambia. The COVID-19 pandemic's recurrence of measles infections and related death highlights serious flaws in the healthcare system that need to be fixed with well-informed policy changes. First, the results emphasize how important it is to create policies that give regular immunization services priority in the event of a public health emergency. Policies should include backup plans that provide funding for vaccination campaigns even in times of emergency, like pandemics. Zambia's Ministry of Health, for example, can establish policies that guarantee vaccinations continue to be an essential part of the emergency response plan. Maintaining sufficient vaccine supply, human resources, and cold chain systems should also be a priority of these policies to avoid disruptions during outbreaks. Second, the results of the study highlight the significance of focused measures to lessen measles outcome disparities, especially for vulnerable groups. Stronger surveillance systems should be the goal of policy to identify high-risk populations and areas with low vaccination rates. For underserved and rural groups, targeted vaccination efforts, such community-based outreach programs, can guarantee fair access to vaccines. These actions are consistent with international frameworks like the WHO's Immunization Agenda 2030, which promotes fair vaccination practices to attain universal coverage [82].

## Strength and limitations

The study has triangulated data from patient’s information collected through the line list during outbreak investigations and mortality counts observed during the 6 year period which is sufficient evidence that the COVID-19 pandemic has affected immunization. The study is not without limitations. We relied on routine data, which depends on data obtained from the routine surveillance. Therefore, case fatality rates could be lower than if the survey had been carried out. Which implies that the mortality burden could be more than what is reported in this study. However, this study closes a significant research gap by offering concrete data on the pandemic's effect on measles mortality and associated predictors emphasizes the necessity of focused public health initiatives to prevent future outbreaks. The results also highlight how crucial it is to increase vaccination rates, strengthening diagnostic and address healthcare inequalities to stop measles-related fatalities in Zambia.

## Conclusion

Measles caused a significant increase in child mortality. Measles mortality was associated with younger age group, vaccination status, testing positive to measles IgM, rush and female sex. The findings suggest that public health interventions focusing on vaccination, rapid detection and appropriate case management are crucial to reducing mortality and preventing further transmission. To achieve population immunity, sustained efforts are required to maintain high coverage rates. To achieve the level of population immunity necessary to prevent the unacceptable high morbidity and mortality rates brought on by measles in susceptible populations, sustained efforts to maintain high coverage rates of the routine first dose of the measles vaccine, along with periodic opportunities for a second dose, are necessary. It is also important to encourage health care providers and caregivers through immunization campaigns to utilize health services during any pandemics so that there is no disruption of immunization, which leads to high morbidity and mortality, especially in children under 5 years. Measles mortality has been significantly impacted by the COVID-19 pandemic, underscoring the critical need for strong and resilient healthcare systems. To meet this challenge, routine immunization services must be restored, catch-up campaigns must be prioritized, and immunization must be integrated with pandemic preparedness strategies. These actions are critical not only to lower measles mortality but also to protect vulnerable populations'health from future public health emergencies.

## Data Availability

The data will be deposited in the figshare online access for easy access for who may wish to look at the data.
